# In Vivo Follow-Up of Gene Inhibition in Solid Tumors Using Peptide-Based Nanoparticles for siRNA Delivery

**DOI:** 10.3390/pharmaceutics13050749

**Published:** 2021-05-19

**Authors:** Isabel Ferreiro, Coralie Genevois, Karidia Konate, Eric Vivès, Prisca Boisguérin, Sébastien Deshayes, Franck Couillaud

**Affiliations:** 1 Imagerie Moléculaire et Thérapies Innovantes en Oncologie—EA 7435 IMOTION, Université de Bordeaux, 33076 Bordeaux, France; isabel.ferreironeira@gmail.com (I.F.); coralie.genevois@u-bordeaux.fr (C.G.); 2 VIVOPTIC TBM-Core, Université de Bordeaux, CNRS UMS 3427, INSERM US 005, 33076 Bordeaux, France; 3 PhyMedExp, Université de Montpellier, Inserm U1046, CNRS UMR 9214, 34395 Montpellier, France; karidia.konate@inserm.fr (K.K.); eric.vives@umontpellier.fr (E.V.); prisca.boisguerin@inserm.fr (P.B.)

**Keywords:** cancer therapy, siRNA, gene silencing, peptide-based nanoparticles, optical imaging

## Abstract

Small interfering RNA (siRNA) exhibits a high degree of specificity for targeting selected genes. They are efficient on cells in vitro, but in vivo siRNA therapy remains a challenge for solid tumor treatment as siRNAs display difficulty reaching their intracellular target. The present study was designed to show the in vivo efficiency of a new peptide (WRAP5), able to form peptide-based nanoparticles (PBN) that can deliver siRNA to cancer cells in solid tumors. WRAP5:siRNA nanoparticles targeting firefly luciferase (Fluc) were formulated and assayed on Fluc-expressing U87 glioblastoma cells. The mode of action of WRAP5:siRNA by RNA interference was first confirmed in vitro and then investigated in vivo using a combination of bioluminescent reporter genes. Finally, histological analyses were performed to elucidate the cell specificity of this PBN in the context of brain tumors. In vitro and in vivo results showed efficient knock-down of Fluc expression with no toxicity. WRAP5:siFluc remained in the tumor for at least 10 days in vivo. Messenger RNA (mRNA) analyses indicated a specific decrease in Fluc mRNA without affecting tumor growth. Histological studies identified PBN accumulation in the cytoplasm of tumor cells but also in glial and neuronal cells. Through in vivo molecular imaging, our findings established the proof of concept for specific gene silencing in solid tumors. The evidence generated could be translated into therapy for any specific gene in different types of tumors without cell type specificity but with high molecular specificity.

## 1. Introduction

Small interfering RNAs (siRNAs) offer new opportunities for cancer therapy since they can control the expression of genes involved in cancer progression. siRNAs can enter the RNA-induced silencing complex (RISC), which then induces enzyme-catalyzed degradation of their complementary messenger RNA, thus disrupting specific molecular pathways with low toxicity [1,2]. siRNAs have a high degree of specificity with respect to their target, far superior to that achieved by most anticancer agents [3], indicating their therapeutic potential as drugs of the future [4]. However, clinical applications of siRNA-based therapy are still limited since naked siRNAs are unstable in the bloodstream, can be immunogenic, and, due to their large molecular weight and highly anionic character, do not cross membranes to enter cells [5,6,7]. Consequently, new delivery strategies have been developed to efficiently get siRNAs to their site of action.

Broad diversity of materials and strategies are currently being explored to address the challenges of in vivo delivery, including polycationic polymers [8], lipids [9], nanoparticles (NPs), and peptide-based formulations [5]. Since 2016, through innovative delivery strategies, there has been an acceleration in the development of oligonucleotides as therapeutics, with the approval of several molecules by the U.S. Food and Drug Administration (FDA). Two therapeutics based on RNA interference (RNAi) have been approved; for instance, ONPATTRO^®^ (Partisiran) for polyneuropathy in hereditary transthyretin-mediated (hATTR) amyloidosis in 2018 [10] and GIVLAARI™ (Givosiran) for acute hepatic porphyria (AHP) in 2019 [11].

Among them, cell-penetrating peptides (CPPs) appear to be one of the most promising non-viral strategies to improve the intracellular routing of large molecules, including siRNAs [12,13,14]. CPPs are short polycationic sequences (less than 35 amino acids) that are amphipathic and are able to cross the plasma membrane and, therefore, translocate different cargoes into cells. Some of them having primary or secondary amphipathic properties can form non-covalent complexes with the siRNA due to electrostatic interactions between the negatively charged backbone of the siRNA and the cationic residues of the CPP. Beyond its neutrality, an excess of CPP leads to the formation of complexes able to self-assemble as positively charged peptide-based nanoparticles (PBNs), which is important for translocation through the cell membrane and for delivering the therapeutics [15,16,17,18].

The recently developed short-size (15 aa) CPP WRAP5 is able to “wrap” the siRNA into efficient PBNs. The PBNs formed to result in a fast translocation into the cell without any cytotoxic effects, displaying twice as much efficiency to knock down the targeted gene as other peptide analogs [19]. Moreover, siRNA-loaded WRAP PBNs are internalized mainly via direct cell membrane translocation, avoiding entrapment in endosomal vesicles acknowledged to reduce their efficiency [20]. WRAP5 is thus a suitable candidate to test the capacity of these CPP:siRNA to enter cells in vivo in the context of a solid tumor and to specifically inhibit the targeted gene.

In this study, we established a proof of concept for specific gene inhibition in vivo using WRAP5:siFluc PBNs by targeting the bioluminescent gene, firefly luciferase (Fluc), in experimental subcutaneous tumors. First, we confirmed in vitro that WRAP5:siRNA PBNs specifically inhibit targeted genes by RNA interference on mRNA. Then, by using bioluminescence imaging (BLI), we showed that WRAP5:siRNA PBNs were able to silence the targeted gene in tumor cells. Using orthotopic brain tumors, we also showed that WRAP5:siRNA PBNs not only entered cancer cells but also the cells of surrounding tissues, demonstrating their great efficiency to penetrate tissues in vivo.

## 2. Materials and Methods

*Plasmid Construction*. CMV promoter and Nluc cDNA from the plasmid pcDNA5-FRT-CMV-Nluc [21] were cloned downstream the Fluc gene in the plasmid pcDNA5-FRT-CMV-Fluc fortin [22]) to generate the pcDNA5-FRT-CMV-Fluc-CMV-Nluc plasmid.

*Cell Line Generation and Culture***.** U87 cell lines (U87 MG, human glioblastoma) were maintained in Dulbecco’s modified Eagle’s medium (Invitrogen, Carlsbad, CA, USA) supplemented with 10% fetal bovine serum (Invitrogen), 1% antimycotic-antibiotic mix (PSA, Invitrogen), and 1% nonessential amino acid (MEM NEAA, Invitrogen). Cell lines were maintained in a humidified 5% CO_2_ incubator at 37 °C. The DNA construct was integrated into the genome by homologous recombination at the Flp Recombination Target (FRT) site (Flp-in system, Invitrogen). The receiver cell line was a clonal U87-FRT cell line containing a unique, already reported, FRT site [22]. Recombination was catalyzed by the Flp recombinase, encoded in the pOG44 vector, and co-transfected with a DNA construct using TransFast™ Transfection Reagent (Promega, Madison, WI, USA). Hygromycin B (50 μg/mL (Euromedex, Souffelweyersheim, France) was used to select the U87-FRT-CMV-Fluc-CMV-Nluc cell line (U87-CMV/Fluc^+^Nluc^+^). The U87-FRT-CMV-Nluc-IRES-Fluc (U87-CMV/Fluc-IRES-Nluc) and U87-FRT-CMV-Fluc (U87-CMV/Fluc^+^) were obtained using the same method as previously described [21,22].

*Nanoparticle formulation and cell transfection procedures.* The peptide-based nanoparticle (PBNs) WRAP5:siRNA were formulated as previously described [19,23]. Briefly, they were formulated in 5% glucose at a peptide:siRNA ratio of R = 20 with a siRNA-targeting Fluc (siFluc: CUUACGCUGAGUACUUCGA (dTdT)) or a siRNA scramble control (siSCR: AGCUUCAUGAGUCGCAUU (dTdT)) (Eurogentec; Seraing, BE), pre-incubated for 30 min at room temperature. For in vivo silencing experiments, siRNA-Fluc-Cy3 and siRNA-Fluc-Alexa790 were added to the formulation (2% of each). For the immunohistochemistry experiment, siRNA-Fluc-Cy3 was added to the formulation (10%). For the in vitro luciferase assay, 5000 cells per well were seeded 24 h before the experiment in 96 well plates. The following day, the growth medium covering the cells was replaced by 70 µL of fresh, pre-warmed serum-free DMEM. WRAP5:siRNA PBN solutions (30 µL at the indicated siRNA concentrations) were added and the plate was incubated for 1 h 30 at 37 °C. Finally, 100 μL of DMEM supplemented with 20% FBS was added to each well without withdrawing the PBNs, and the cells were then incubated for another 36 h at 37 °C. The experimental procedure was designed to test WRAP5:siRNA PBNs at a peptide:siRNA molar ratio of R = 20, containing siRNA concentrations of 5, 10, 20, or 50 nM in the final volume of 200 µL.

*Animal handling and tumor generation.* Animal manipulations were performed in line with French and European directives on the care and use of animals, which were approved by the local ethics committee (CEEA 50) under agreement APAFIS#8810. Immunodeficient NSG (NOD/SCID/IL-2Rγnull) mice between 8 and 16 weeks old were reared at the University of Bordeaux animal facility. The mice were maintained in standard conditions under a 12-h light/dark cycle with water and food provided ad libitum. Animal manipulations were performed on anesthetized mice using 2% isoflurane (Belamont, Nicholas Piramal Limited, London, U.K.) in the air. The regions of the mice to be imaged were previously shaved with clippers and depilatory cream. Subcutaneous tumors were generated by injection of U87-CMV/Fluc^+^Nluc^+^ cells (2·10^6^/100 μL PBS) on the left posterior leg of the mouse. For the brain tumor implantation, anesthetized mice were placed in a stereotaxic frame. U87-CMV/Fluc^+^ cells (1·10^6^/5 μL PBS) were implanted in the right parietal brain area, 1 mm posterior and 2.5 mm lateral to the bregma skull, at a depth of 2.5 mm from the skull surface. Subcutaneous and brain tumor growth was monitored by bioluminescence imaging. WRAP5:siRNA PBNs solutions were injected into the subcutaneous tumor (20 µg/20 µL) or into the orthotopic tumor using a stereotaxic frame (5 µg/5 µL).

*In vitro luciferase activity measurement*. For Fluc and Nluc activity measurement, U87 cells were incubated in a passive lysis buffer (50 µL, Promega) for 15 min at room temperature with shaking. The cell lysate (5 µL) was mixed with Luciferase Assay Substrate (50 µL; Promega) to measure Fluc activity. Nluc activity was measured using 50 µL of the Nano-Glo™ reagent (Promega) and 1 µL of cell lysate. Luminescence activities were measured 10s after mixing lysate and substrate using a luminometer (LUMAT 9501; Berthold Technology, Bad Wildbad, DE) and expressed in relative light units (RLU).

*mRNA extraction and quantitative RT-PCR.* Total mRNA was isolated from U87 subcutaneous tumors (30 mg) using a NucleoSpin RNA kit (Macherey-Nagel, Hoerdt, France) according to the manufacturer’s instructions. Total RNA was quantified with a Nanodrop (Thermo Fisher Scientific, Waltham, MA, USA). cDNA synthesis was performed on 500 ng of total RNA using ImProm-II^TM^ Reverse Transcription System (Promega) according to the manufacturer’s protocol. Quantitative PCR was performed using Maxima SYBR/Green/Fluorescein qPCR Master Mix (Invitrogen, Carlsbad, CA, USA) with MyIQ Quantitative thermocycler (Bio-Rad, Hercules, CA, USA) according to the manufacturer’s instructions. Primers (Eurogentec; Seraing, Belgium) for Fluc amplification were Fluc-Fw (5′-TCCATTCCATCACGGTTTTGG-3′) and Fluc-Rv (5′-GCTATGTCTCCAGAATGTAGC-3′), while the primers for GAPDH amplification were GAPDH-Fw (5′-GCCAAGGTCATCCATGACAAC-3′) and GAPDH-Rv (5′-GAGGAGTGGG TGTCGCTGTTG-3′). Reactions were run in triplicate in three independent experiments. Expression data were normalized to the geometric mean of housekeeping human gene GAPDH to control variability in expression levels. They were then analyzed using the 2^DDCt^ method.

*In vivo bioluminescence imaging (BLI) and fluorescence reflectance imaging (FRI).* BLI and FRI were performed at Vivoptic platform (Univ. Bordeaux, CNRS, INSERM, TBM-Core, UMS 3427, U.S. 5, F-33000 Bordeaux) using a Lumina LT system (PerkinElmer, PerkinElmer Inc., Boston, MA, USA). To perform BLI, mice received an intraperitoneal injection of d-luciferin (Promega, 2.9 mg in 100 µL PBS) or Nano-Glo™ substrate containing furimazine (20 μg in 100 μL sterile PBS, Promega) for Fluc or Nluc analysis, respectively. The mice were sedated 5 min after the substrate injection. Bioluminescence images (1 min, binning 4 × 4) and photographs (100 ms) were taken successively at 8 min. For dual imaging, Fluc and Nluc were measured sequentially with a 6 h delay. For the orthotopic tumors, the brain was removed from the euthanized mice, placed in cold phosphate-buffered saline (PBS) on a glass slide, and imaged. In vivo FRI was performed using an excitation filter at 745 nm and fluorescence emission was detected with the 810–875 nm filter. The ex vivo brain tumor was imaged using an excitation filter at 535 nm and 575–650 nm emission filter. Bioluminescence and fluorescence images were analyzed using Living Image software, and the signals were quantified by placing a region of interest (ROI) on the tumor.

*Immunohistochemistry and microscopy.* Brains with tumors were embedded in OCT medium (CellPath, Newtown, UK), frozen, and stored at −80 °C. Cryosections (10 µm) were obtained and fixed 10 min with 4% of paraformaldehyde (PFA, Deltamicroscopies, Mauressac, France). Slices were incubated with a rabbit anti-GFAP (diluted 1/1000^e^, Dako, Les Ulis, France), a mouse anti-Neun (diluted 1/100^e^, MerckMillipore, Molsheim, France), or a mouse anti-human CD44 (diluted 1/100^e^, BD Biosciences, San Jose, CA, USA) at 4 °C overnight. Sections were then incubated with a goat anti-rabbit or a goat anti-mouse, both conjugated to Alexa647 (diluted at 1/500^e^, Invitrogen) for 1 h at room temperature. The nuclei were stained with DAPI (Invitrogen), and slices were finally mounted in Fluoromount medium (Deltamicroscopies). Images were obtained using a confocal Leica DM6 CFS TCS SP8 microscope (Leica, Wetzlar, Germany). DAPI detection was performed using the 405 nm laser, while cyanine 3 detection was performed using the 552 nm laser, and Alexa647 detection was performed using the 638 nm laser.

*Statistical analyses.* Comparisons were performed between two groups using a Student‘s *t*-test. *P* values of less than 0.05 were considered statistically significant. The calculations were performed using GraphPad Prism software.

## 3. Results

### 3.1. WRAP5:siRNA PBNs Selectively Silenced the Target Gene by RNA Interference In Vitro

**Figure 1:**
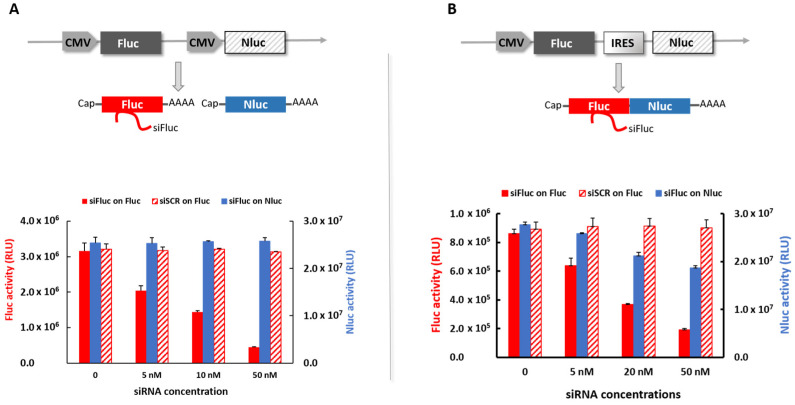
In vitro silencing of Fluc and Nluc in genetically modified U87 cell lines by WRAP5:siFluc and WRAP5:siSCR PBNs. (**A**) On the U87-CMV/Fluc+Nluc+ cell line with 2 distinct mRNAs. Schematic representation of the dual-luciferase reporter plasmid and quantitative analysis of the luciferase activities (Fluc and Nluc) after treatment with PBNs WRAP5:siFluc (red full histogram bars) or WRAP5:siSCR (hatched histogram bars). (**B**) On the U87-CMV/Fluc-IRES-Nluc cell line with a common mRNA. Schematic representation of the dual-luciferase plasmid and quantitative analysis of the luciferase activities after treatment with PBNs WRAP5:siFluc (red full histogram bars) or WRAP5:siSCR (hatched histogram bars). In both models, cells were treated with the siFluc, and the Nluc activity was measured (blue full histogram bars). The data represent the mean ± SD (*n* = 4).

WRAP5:siFluc PBNs have previously been characterized for Fluc inhibition in the U87 cell line [19]. WRAP5 was shown to spontaneously self-assemble with siFluc to form WRAP5:sFluc nanoparticles. Usually formulated in 5% glucose and at a peptide:siRNA molar ratio of R = 20, WRAP5:siFluc PBNs were identified as smaller globular nanoparticles with a mean size of 80.0 ± 4.9 nm, a positive zeta potential of 28.8 ± 0.9 mV, and a polydispersity index of 0.29 ± 0.05 relevant of homogenous and monodisperse distribution [19]. To further demonstrate that WRAP5:siFluc acted via an RNA interference mechanism, the dual-luciferase reporter cell line, U87-CMV/Fluc^+^Nluc^+^ was used. This cell line was genetically modified to overexpress the firefly luciferase (Fluc) and the Nano luciferase (Nluc), both under transcriptional control of their own constitutive promoter, Cytomegalovirus (CMV), leading to 2 different mRNAs (see diagram in Figure 1A). This cell line allowed us to perform the proof of concept of the PBNs cellular internalization by easily measuring the decrease in Fluc expression while using Nluc as an internal control for normalization. Cells were treated with WRAP5:siFluc or its scrambled version (WRAP5:siSCR) and the 2 luciferases’ activities were measured. As shown in Figure 1A, Fluc activity decreased in a dose-dependent manner after treatment with increasing doses of WRAP5:siFluc (red full histogram bars), while the Nluc expression remained constant (blue full histogram bars). Fluc activity was not inhibited by WRAP5:siSCR (hatched histogram bars), showing that Fluc inhibition was not due to the toxicity of the chemical composition of WRAP5:siRNA but was linked to the oligonucleotide sequence.

To verify that the Fluc silencing observed in our previous figure occurred through a specific siRNA mechanism, the U87-CMV/Fluc-IRES-Nluc cell line was used. This cell line was genetically modified to express a bicistronic vector, including a cap-dependent translation of Fluc and an IRES-dependent translation of Nluc, under transcriptional control of the same constitutive CMV promoter (see diagram in Figure 1B). In this setup, the two luciferase genes were transcribed as a single mRNA molecule, but their translation was independent. As shown on the graph in Figure 1B, Fluc and Nluc activities (full histogram bars) were both affected by treatment with WRAP5:siFluc PBNs in a dose-dependent manner. As Fluc and Nluc were transcribed on the same mRNA, the siRNA mechanism due to the WRAP5:Fluc PBNs affected the activities of both luciferases. Differences in the time courses were due to differences in half-life between Fluc and Nluc [24,25]. Treatment with WRAP5:siSCR PBNs (hatched histogram bars) did not affect Fluc activity, indicating the absence of toxicity of the PBN formulation. These results suggest that the RNA interference mechanism is probably due to mRNA degradation.

### 3.2. Specific Inhibition of Gene Expression in In Vivo Xenograft Mouse Models of GBM

**Figure 2:**
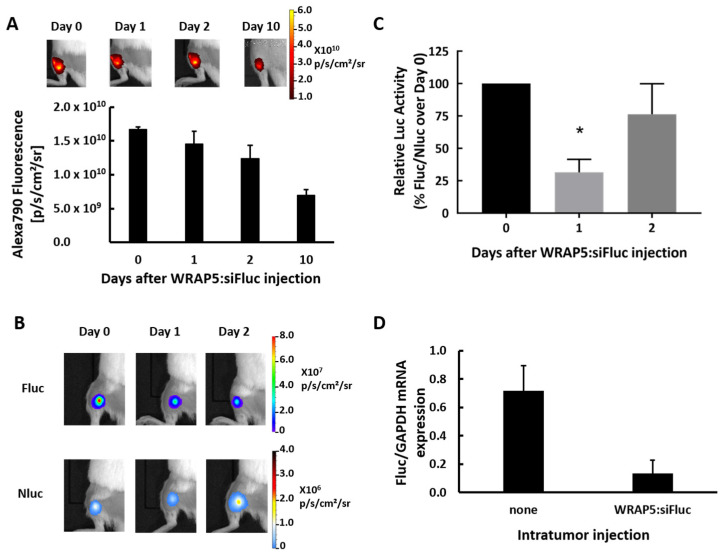
In vivo Fluc silencing in a tumor xenograft mouse model. (**A**) Representative images and quantification of the FRI signal in the tumor xenografts over time after intra-tumoral injection of WRAP5:siFluc-Alexa790 PBNs (*n* = 4). (**B**) Representative dual bioluminescent imaging (BLI) of Fluc and Nluc expression at days 0, 1, and 2 after injection of WRAP5:siFluc PBNs (20 µg) into U87 xenograft tumors (*n* = 11). (**C**) Quantitative analysis of Fluc activity over Nluc on days 0, 1 and 2 after injection of WRAP5:siFluc PBNs (Student′s *t*-test, * *p* < 0.05; *n* = 11). (**D**) Quantitative analysis of Fluc mRNA expression into U87 tumors xenografts non-injected and 2 days after WRAP5:siFluc (20 µg) injection (*n* = 4). Luciferase mRNA level is expressed as a ratio with GAPDH (FLuc/GAPDH).

To assess the therapeutic potential of WRAP5 as siRNA delivery carriers, we conducted in vivo gene silencing of the Fluc gene in mice bearing U87 xenografts. To this end, the U87-Fluc^+^Nluc^+^ cell line was subcutaneously transplanted into the back limbs of the mice to establish tumors. First, the fate of the PBNs in the tumor after injection was monitored by injecting WRAP5:siFluc PBNs in which the siRNA was labeled with a fluorophore (WRAP5:siFluc-Alexa790), and the fluorescence was tracked for 10 days with IVIS Lumina (Figure 2A). We observed that fluorescence from the fluorophore Alexa790 attached to the siFluc decreased slowly over time, with a 50% reduction 10 days after the injection.

The constitutive expression of Fluc and Nluc allowed tumor growth to be monitored in vivo by bioluminescence imaging (BLI). When the tumor had reached a minimum of 2 × 10^6^ p/s, the mice were assayed for gene silencing. They were treated by intratumoral injection of WRAP5:siFluc-Alexa790 PBNs (20 µg siRNA; glucose media 5%), and the Fluc and Nluc activities were evaluated at day 1 and day 2 after injection by measuring the BLI intensities (N = 11) (Figure 2B). Treatment with the WRAP5:siFluc PBNs resulted in a decrease in Fluc intensity on day 1, while the Nluc signal gradually increased over time, correlating with tumor growth. Quantification of the Fluc expression over Nluc is shown at different time points (Figure 2C), and a statistically significant reduction in Fluc expression (−70%) was observed 1 day after injection (* *p* < 0.05 vs. day 0).

In order to confirm that the Fluc silencing observed in vivo was due to specific mRNA Fluc degradation mediated by the siRNAs as previously demonstrated, mRNA levels of Fluc were quantified by reverse transcription (RT)-PCR into non-injected and WRAP5:siFluc PBNs injected tumors (N = 4 for each). We observed a significant reduction in mRNA Fluc levels relative to human GAPDH 2 days after treatment with WRAP5:siFluc PBNs (Figure 2D). These results correlate well with the aforementioned in vitro data (Figure 1), showing that WRAP5:siRNA PBNs acted by degrading Fluc mRNA.

### 3.3. In Vivo Uptake of WRAP5:siFluc PBNs and Cell Tropism of WRAP5:siFluc PBNs

**Figure 3:**
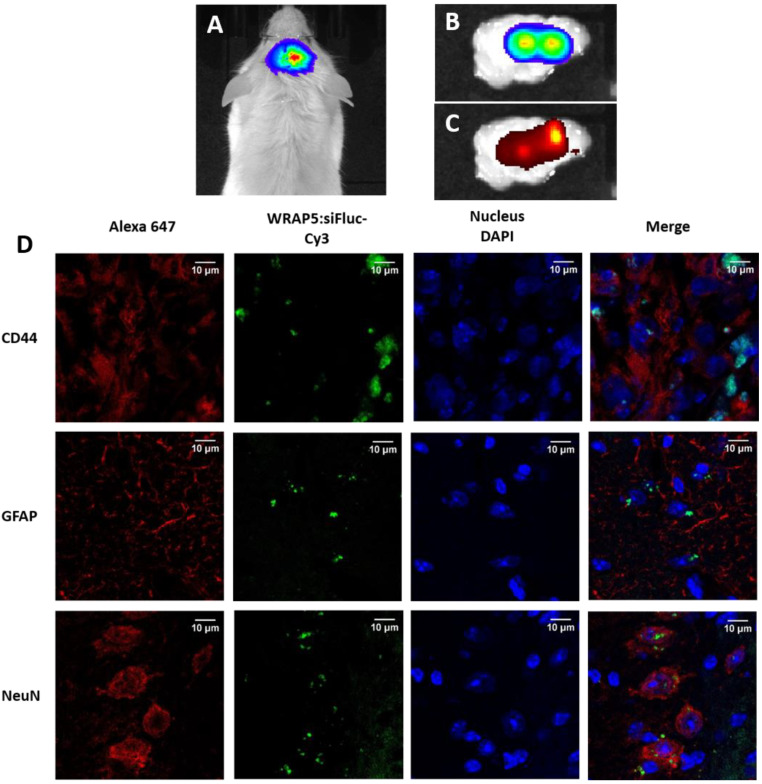
Cellular uptake of WRAP5:siFluc-Cy3 PBNs on U87 orthotopic tumor. (**A**) Bioluminescence imaging (BLI) of a mouse bearing an orthotopic tumor. (**B**,**C**) BLI and FRI, respectively of the excised brain 8 h after WRAP5:siFluc-Cy3 PBNs injection. (**D**) Immunofluorescence images on cryosections (10 µm). The proteins GFAP, NeuN, and CD44 revealed with an Alexa647 secondary antibody are shown in red. The WRAP5-siFluc attached to the fluorophore Cy3 is shown in green, and the nucleus stained in blue with DAPI.

To evaluate the in vivo uptake of PBNs in the mouse tumor and their tropism in the different brain cell types, immunohistochemically (IHC) analyses were performed in orthotopic U87 mouse models treated with PBNs. U87-CMV/Fluc^+^ cells were injected into the right parietal brain area of NSG mice to establish tumors. Tumor growth was monitored by BLI (Figure 3A), and when the tumors reached a minimum of 3 × 10^7^ photons/s, WRAP5:siFluc-Cy3 PBNs were injected into the brain tumor. After 8 h, the brain was excised, imaged by BLI that revealed the tumor (Figure 3B) and by FRI for the PBNs’ localization (Figure 3C), which were snap-frozen for further histological analyses. To evaluate the cellular uptake and the potential diffusion of PBNs following injection, the expression of different protein markers of each cell type was studied by immunohistochemistry (IHC) (Figure 3D). The anti-glial fibrillary acidic protein (GFAP) antibody was used as a marker of glial cells (mouse glial cells and U87 cells), the anti-neuronal nuclei (NeuN) to mark the neurons, and the human anti-CD44 was used as a cancer cell marker. As expected, PBNs (in green) were localized in tumor cells revealed by a human CD44 antibody (red). IHC analyses also showed that some PBNs (green) were colocalized with GFAP and NeuN markers (both red), revealing the mobility of the PBNs after injection.

## 4. Discussion

Generation of peptide-based nanoparticles (PBNs) by mixing siRNA to small amphipathic peptides offers many physical and biological advantages compared to naked siRNA [17,26]. The encapsulated siRNAs are less sensitive to nucleases, and the peptide component of the PBNs improved cellular internalization. CPPs were shown not to impair siRNA efficiency on cultured cells, and several CPP:siRNA were reported to exhibit efficient gene silencing in vitro for doses of around 20 nM of siRNA [19,23]. However, like many other nanoparticles, CPP:siRNA failed to reach cancer cells efficiently in vivo, first, because a very weak percentage of blood-injected dose reached the tumor, and second, because within the tumor, a very small number of molecules reached their intracellular target [27].

In the present study, siRNAs were associated with a new cell-penetrating peptide called WRAP5 that has been reported to induce efficient gene silencing on cultured cells [19]. The reporter gene, Fluc, was chosen as the molecular target to monitor in vivo the interaction of siRNA with its target by BLI. It has previously been shown that WRAP5:siFluc PBNs inhibited Fluc activity in vitro without cytotoxicity [19]. The PBNs were expected to interact with Fluc mRNA through the RISC machinery in the cancer cell cytoplasm, stopping the mRNA translation and inducing its degradation. The mode of action at the molecular level was confirmed in vitro using two different dual-luciferase reporter cell lines. In the cell line with both the luciferases, Fluc and Nluc, encoded by two distinct mRNA, PBNs only silence Fluc mRNA, decreasing Fluc activity, while no change was observed in Nluc activity (Figure 1). In the cell line with Fluc and Nluc encoded on the same mRNA, interaction of siFluc with Fluc mRNA affected both Fluc and Nluc activity. Fluc and Nluc were translated by two distinct mechanisms, namely, the cap-dependent translation and the IRES-dependent translation for Fluc and Nluc, respectively. Hence, Nluc inhibition by a siRNA-targeting Fluc was more likely due to degradation of the common mRNA impairing translation of the two cistrons. Taken together, these data confirm PBNs reduce the BLI signal in Fluc-expressing cells by decreasing Fluc mRNA level by a highly specific mechanism of RNA interference.

For the in vivo experiment, the cell line with Fluc and Nluc encoded by two distinct mRNA was used to generate subcutaneous tumors in mice. To ensure sufficient concentration in the tumor and to avoid massive trapping by the liver when injected intravenously, WRAP5:siFluc PBNs were injected directly into the tumor. As PBNs targeted Fluc, BLI allowed the gene silencing to be monitored in vivo. The bioluminescence signal from Fluc significantly decreased 1 day after the PBN injection, but the decline was limited and transient as the BLI signal partially recovered 2 days after injection (Figure 2B). The decline in the Fluc signal was due to a decrease in Fluc mRNA content in the tumor, as confirmed by qRT-PCR (Figure 2D), further supporting the in vitro data and showing that siFluc acted by degradation of the Fluc mRNA induced by RNAi. As expected, the BLI signal from Nluc was not affected (Figure 2B), further demonstrating that the high molecular specificity of the WRAP5:siFluc tool was preserved in vivo.

The fluorescence signal slowly decreased over time, but about half of the fluorescence signal remained in the tumor 10 days after the PBNs injection. From fluorescence images assuming an almost spherical shape, the volume of diffusion could be calculated for a tumor of around R = 3 mm. As the PBN injection contained 1.45 nmol of siFluc, the estimated concentration is about 13 µM in the tumor area, around 650 times higher than the efficient dose for in vitro inhibition (20 nM). Thus, the limited and transient inhibition of Fluc activity may be attributed to several parameters, including the low efficiency of WRAP5:siFluc PBNs to reach the target cells. As already reported in solid tumors for many types of nanoparticles, the majority of intratumoral nanoparticles were either trapped in the extracellular matrix or taken up by tumor-associated macrophages [27]. For instance, only 2% of extravased folic acid-coated gold and silica nanoparticles were reported to reach cancer cells in solid tumors [27]. Present data on brain tumors further support the trapping hypothesis as the distribution of fluorescence associated with siFluc was not homogenous in the tumors and appeared as high-intensity spots on histological sections. Furthermore, at the cellular level, the fluorescence signal was somewhat weak, further supporting the hypothesis that very few siFluc molecules reached the target cells. Alternatively, and not tested in the present work, the transient nature of the BLI signal decrease may be due to the rapid degradation of PBNs in the tumor.

Fluorescence labeling of the siFluc enabled the WRAP5:siRNA PBNs distribution in the mouse to be monitored by fluorescence imaging, showing that fluorescence remained in the tumor area and did not diffuse over a long distance (Figure 2A). As the fluorescent label is linked to the siRNAs, the fluorescence detected in the tumors might be attributed to either remaining WRAP5:siFluc-Alexa790 PBNs, or to siFluc-Alexa790 PBNs released from WRAP5 or to free Alexa790 label resulting from siRNA degradation. Furthermore, linked and free Alexa790 could be either intracellular or extracellular. The persistence of the fluorescence signal up to 10 days in the tumor did not imply that siRNAs remain functional. Additional experiments are needed to gain a comprehensive picture of the situation in the tumor regarding the fate of siRNA in time and its concentrations in each tumor and cell compartments. Altogether, the present data demonstrate that inhibition of a molecular target is possible in vivo using WRAP5:siRNA PBNs, but the reasons for the somewhat low efficiency need to be further explored.

WRAP5:siFluc PBNs are expected to facilitate cell penetration for siRNAs, but PBNs are not designed and functionalized to enter a specific cell type or to target a specific tissue. This was confirmed by the injection of PBNs into brain tumors (Figure 3). WRAP5:siFluc entered the cancer cell (CD44+) in line with a decrease in the BLI signal observed in subcutaneous tumors. Fluorescence was also detected in glial cells (GFAP+) without discrimination between the U87 tumor cells and the host glial cells. Finally, fluorescence was also detected in some neurons (NeuN+). As histology was performed 8 h after the PBNs injection, the fluorescence signal was expected to be associated with the WRAP5:siRNA-Fluc PBNs complex.

## 5. Conclusions

The current data demonstrate the in vivo ability of WRAP5:siRNA PBNs to inhibit a molecular target in a tumor. While the PBN entered different cell types, it interacted specifically with Fluc mRNA, thus decreasing mRNA content and luciferase activity in the tumor, as revealed by BLI. These results lead to the proof of concept in vivo for targeting specific genes in cancer cells using PBN as an siRNA vehicle. It provides a novel therapeutic option for future development in the clinic of a highly specific drug, targeting master regulatory genes in solid tumors.

## Data Availability

Not applicable.
